# Biorefining Potential of Wild-Grown *Arundo donax*, *Cortaderia selloana* and *Phragmites australis* and the Feasibility of White-Rot Fungi-Mediated Pretreatments

**DOI:** 10.3389/fpls.2021.679966

**Published:** 2021-07-02

**Authors:** Ricardo M. F. da Costa, Ana Winters, Barbara Hauck, Daniel Martín, Maurice Bosch, Rachael Simister, Leonardo D. Gomez, Luís A. E. Batista de Carvalho, Jorge M. Canhoto

**Affiliations:** ^1^Centre for Functional Ecology, Department of Life Sciences, University of Coimbra, Coimbra, Portugal; ^2^Molecular Physical-Chemistry R&D Unit, Department of Chemistry, University of Coimbra, Coimbra, Portugal; ^3^Institute of Biological, Environmental and Rural Sciences, Aberystwyth University, Aberystwyth, United Kingdom; ^4^Centre for Novel Agricultural Products, Department of Biology, University of York, York, United Kingdom

**Keywords:** *Arundo donax*, biomass, cell wall, *Cortaderia selloana*, marginal lands, *Miscanthus* × *giganteus*, *Phragmites australis*, white-rot fungi

## Abstract

*Arundo donax*, *Cortaderia selloana* and *Phragmites australis* are high-biomass-producing perennial Poalean species that grow abundantly and spontaneously in warm temperate regions, such as in Mediterranean-type climates, like those of Southern Europe, Western United States coastal areas, or in regions of South America, South Africa and Australia. Given their vigorous and spontaneous growth, biomass from the studied grasses often accumulates excessively in unmanaged agro-forestry areas. Nonetheless, this also creates the demand and opportunity for the valorisation of these biomass sources, particularly their cell wall polymers, for biorefining applications. By contrast, a related crop, *Miscanthus* × *giganteus*, is a perennial grass that has been extensively studied for lignocellulosic biomass production, as it can grow on low-input agricultural systems in colder climates. In this study Fourier transform mid-infrared spectroscopy (FTIR), high-performance anion-exchange chromatography (HPAEC) and lignin content determinations were used for a comparative compositional characterisation of *A*. *donax*, *C*. *selloana* and *P*. *australis* harvested from the wild, in relation to a trial field-grown *M*. × *giganteus* high-yielding genotype. A high-throughput saccharification assay showed relatively high sugar release values from the wild-grown grasses, even with a 0.1M NaOH mild alkali pretreatment. In addition to this alkaline pretreatment, biomass was treated with white-rot fungi (WRF), which preferentially degrade lignin more readily than holocellulose. Three fungal species were used: *Ganoderma lucidum*, *Pleurotus ostreatus* and *Trametes versicolor*. Our results showed that neutral sugar contents are not significantly altered, while some lignin is lost during the pretreatments. Furthermore, sugar release upon enzymatic saccharification was enhanced, and this was dependent on the plant biomass and fungal species used in the treatment. To maximise the potential for lignocellulose valorisation, the liquid fractions from the pretreatments were analysed by high performance liquid chromatography – photodiode array detection – electrospray ionisation tandem mass spectrometry (HPLC-PDA-ESI-MS^*n*^). This study is one of the first to report on the composition of WRF-treated grass biomass, while assessing the potential relevance of breakdown products released during the treatments, beyond more traditional sugar-for-energy applications. Ultimately, we expect that our data will help promote the valorisation of unused biomass resources, create economic value, while contributing to the implementation of sustainable biorefining systems.

## Introduction

Horizon 2020, a European Union (EU) research and innovation framework programme has generously funded research toward a sustainable bio-based economy. This is a recognition of the importance of reducing the dependency on fossil fuels in Europe, and a substantial contribution to the EU’s ambitious climate and energy aims for 2030, which includes an EU-wide target for renewable energy of at least 27% of final energy consumption (European Comission, 2017).

Lignocellulosic biomass and its main constituent cell wall polymers represent the most abundant renewable resource on Earth (Pauly and Keegstra, 2008). Biomass can be derived from dedicated biomass crops, such as perennial herbaceous crops, which are being evaluated as biomass feedstocks throughout the world (Zegada-Lizarazu et al., 2010). Factors such as low production cost, fast-growth, high biomass production, relative low water and nutrient requirements account for the advantage of using these crops as feedstocks for second generation biorefineries (Zegada-Lizarazu et al., 2010; Alzagameem et al., 2019). One of these herbaceous crops is *Miscanthus* × *giganteus* J.M.Greef, Deuter exHodk., Reuvoize, a vigorous inter-specific hybrid between *M*. *sinensis* and *M*. *sacchariflorus*, which has been well researched in terms of its use as a dedicated lignocellulosic crop in Europe (Lewandowski et al., 2000; Heaton et al., 2008; Clifton-Brown et al., 2016; da Costa et al., 2019). Dedicated biomass crops do, however, raise concerns related to land use competition against food production or long-term soil health (Mitchell et al., 2016). Nonetheless, high quantities of biomass from perennial grasses are accumulated from wild vegetation. The abandonment of rural landscapes throughout the 20^*th*^ century has led to the emergence of many derelict, underused and abandoned spaces, which in turn are colonised by vegetation traditionally not considered of great use (Millard, 2004). In this study, the term “marginal land” refers to these types of spaces, while “spontaneous” refers to the vegetation that emerges without the need for cultivation. In the *Centro* Region of Portugal, *Arundo donax* L. (giant cane), *Cortaderia selloana* (Schult. and Schult.f.) Asch. and Graebn. (Pampas grass) and *Phragmites australis* (Cav.) Trin. ex Steud. (common reed), are three abundant and spontaneous grass species, which remain unharvested or become agroforestry waste, squandering potential opportunities for economical gain, and creating land and waste management issues.

Lignocellulose is a highly attractive material for bio-based applications such as fermentation processes to produce a wide range of industrial relevant products. Three groups of polymers constitute lignocellulosic biomass: cellulose, lignin and hemicelluloses. For the latter, in grass cell walls, the most abundant hemicelluloses are arabinoxylans (Carpita, 1996). Besides cellulosic ethanol (Liu et al., 2019), other potential products from cellulose bioconversion include biogas via fermentation with anaerobic bacteria (Cheng et al., 2010), and butyrate and acetate as by-products of the hydrogen fermentation (Pan et al., 2010). Non-biofuel related products such as lactic, citric, acetic, and succinic acids, may also be produced from cellulose fermentation (Watanabe et al., 1998; Ravinder et al., 2001; Kim et al., 2004; Mussatto et al., 2008). Although the bioconversion of hemicelluloses presents its own set of challenges, there is also a range of products that may be derived from this fraction. Examples include the use of pentoses in hemicellulose hydrolysates for ethanol fermentation (Silva et al., 2010), acetone-butanol-ethanol fermentation (Qureshi et al., 2010a, b), or even for xylitol production via fermentation of xylose (Mussatto and Roberto, 2004). Moreover, processing breakthroughs have created the opportunity to dramatically increase the transformation of lignin to value-added products, by improving the yield of low molecular weight aromatic monomers with potential industrial value (Ragauskas et al., 2014; Nguyen et al., 2018; Song et al., 2018). One of these strategies relies on lignin-first biomass fractionation, which uses mild fractionation approaches to prevent lignin recondensation, ensuring a wider range of applications (Renders et al., 2017; Korányi et al., 2020).

Nonetheless, efficient biorefinery conversion of lignocellulosic biomass is hampered by an intrinsic recalcitrance to enzymatic degradation, an evolutionary adaptation aimed at resisting biotic attacks and abiotic stress (Alam et al., 2019). The main cause of this recalcitrance is cell wall complexity and architecture (McCann and Carpita, 2015; Park and Cosgrove, 2015). This results in very high conversion processing costs, making it essential to develop improved biomass processing technologies for optimal biorefining of lignocellulosic biomass.

A commonly employed approach to reduce biorefinery costs is to apply biomass pretreatments which are conducted up-stream in the biomass processing pipeline to enhance the efficiency of down-stream enzymatic hydrolysis and fermentation processes (Mosier et al., 2005). Among diverse pretreatments that have been characterised, mild alkali conditions are particularly promising, as they primarily break ester bonds that cross-link polysaccharides with each other and with lignin, thereby making cellulose more accessible to hydrolytic enzymes (Hendriks and Zeeman, 2009; Xu et al., 2012; Li et al., 2013; Wyman et al., 2013).

The pretreatment of biomass with white-rot fungi (WRF) represents another mild method approach to biomass fractionation, which may allow an increase in the recovery of fermentable sugars, the isolation of reactive lignins and the release of valuable small molecules (Salvachúa et al., 2011; Sun et al., 2011). Biological pretreatments have been less studied than thermochemical ones, possibly because industry often finds slower processing rates unattractive. However, this problem can be addressed by continuous flow processing systems (Scott et al., 1998). Lignin polymers represent the main barrier to degradation due to their large, stereo-irregular structures, and the presence of inter-unit carbon-carbon and ether bonds. Lignin degradation mechanisms require oxidative rather than hydrolytic processes, and ligninolytic agents must have wide substrate specificity and act synergistically. WRF have developed ligninolytic enzymatic machineries, including a wide range of peroxidases and laccases (Ruiz-Dueñas and Martínez, 2009; Dashtban et al., 2010; López et al., 2017). These diverse enzymatic pools allow WRF to deal with different compositional and structural aspects and depolymerise lignin.

**FIGURE 1:**
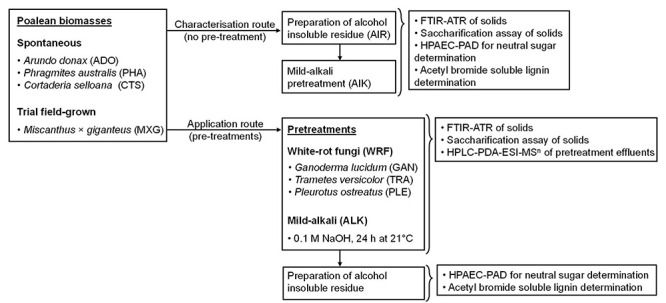
Schematic diagram of the employed experimental study design.

The primary focus of our work was to assess the biorefinery potential of wild-grown spontaneous grass biomass from marginal lands and characterise the mechanism of action of innovative processing methodologies, by combining chemical and biological approaches (Figure 1). For biomass pretreatment, we employed three WRF species, which preferentially degrade lignin rather than holocellulose (Taniguchi et al., 2005; Abidi et al., 2014; López et al., 2017): *Pleurotus ostreatus*, *Ganoderma lucidum* and *Trametes versicolor*. These were applied alone or in combination with a mild alkali pretreatment. The purpose of this approach was to determine whether these would act synergistically on the biomass and release potentially valuable molecules, while reducing recalcitrance. Subsequently, a multidimensional analytical approach, followed by biomass conversion assays was employed. We consider that by identifying economic opportunities for spontaneous vegetation valorisation, landowners would have a monetary incentive to cull excessive vegetation more frequently and employ more efficient land management practices.

## Materials and Methods

### Poalean Lignocellulosic Biomass

Biomass from 3 wild-grown spontaneous grasses was collected at two locations in Central Portugal. Montemor-o-Velho (40.171679°N, 8.671122°W): *Arundo donax* (ADO), *Phragmites australis* (PHA). Serra da Boa Viagem (40.186115°N, 8.903903°W): *Cortaderia selloana* (CTS). Additionally, biomass from *Miscanthus* × *giganteus* (MXG; genotype Mb311) was harvested from a field trial near Aberystwyth, United Kingdom (52.437848°N, 4.026688°W). Sampling for the Portuguese-harvested biomass was done during later summer (September), whereas *M*. × *giganteus* was harvested by late May. All samples consisted in whole tillers cut at soil level from non-senesced plants. For each species, to account for the heterozygosity of the biomass, two biological replicates were collected. The choice of two biological replicates was made to ensure the feasibility of the study. All seed, seed-bearing or flowering structures, were discarded when present. Within a maximum of 5 h from collection, all samples were stored at −80°C until being freeze-dried. Once dry, stems were separated from leaves (including sheath), individual organs were ground using a Retsch SR3 Rotor Beater Mill and passed through a perforated plate screen containing 2 mm diameter holes.

### Preparation of Alcohol Insoluble Residue

A procedure based on a protocol reported by da Costa et al. (2020), was carried out to produce the alcohol insoluble residue (AIR) used in the subsequent analyses. For each sample, approximately 1 g of ground plant biomass was extracted sequentially as follows: with 30 mL 70% (v/v) aqueous ethanol, first for 12 h and then twice more for 30 min in a shaking incubator set at 40°C/150 rpm; three times with 20 mL chloroform/methanol (1:1 v/v), for 30 min incubation at 25°C and 150 rpm; and finally, three times with 15 mL acetone, for 30 min, at 25°C/150 rpm. Between each step of the extraction, the material was collected by centrifugation at 25000 × *g* for 10 min and the supernatants were discarded. Following the third acetone step, the samples were left to dry overnight in a fume hood.

### Inoculum Preparation and Fungal Pretreatment

*Ganoderma lucidum* (GAN), *Trametes versicolor* (TRA) and *Pleurotus ostreatus* (PLE) were used as white-rot fungi (WRF) for biological pretreatments. As described elsewhere (Paiva De Carvalho et al., 2019), morphological examination and molecular analysis, targeting internally transcribed spacer (ITS) regions, allowed the identification of the fungal species used in this study. Fungal inocula were prepared by culturing the individual WRF strains at 23°C on 2.9% potato dextrose agar (PDA, Oxoid CM0139, Basingstoke, England). After 10 days of growth, for each of the three WRF strains, inoculation discs (Ø = 10 mm) were taken from actively growing mycelium on the PDA plates and used to inoculate each sample of the poalean lignocellulosic feedstocks (2 discs per sample), under solid state fermentation (SSF) conditions. To serve as solid media for WRF growth, the grass biomass was prepared as follows: approximately 1.5 g of previously dried and milled but not organic solvent-washed biomass was added to 5 mL deionised water and autoclaved in glass culture tubes capped with hydrophobic cotton. This was performed for each combination of the 3 WRF species, and leaf or stem from the 4 grass species. Additionally, non-inoculated biomass samples (non-WRF treated, NF controls) with an equal volume of deionised water added, were included to serve as negative controls. All cultures were incubated statically at 23°C in the dark for 30 days; with a total of 64 duplicated samples: 4 grass species × 2 organs (leaf or stem) × 4 treatments (3 WRF species plus control). WRF-pretreatment methodologies were adapted from procedures reported elsewhere (Salvachúa et al., 2011; Sun et al., 2011). After incubation, the inoculation discs were removed, 5 mL deionised water were added, and the samples were thoroughly mixed and incubated at 30°C for 24h with constant mixing. Samples were then centrifuged (2000 × *g* for 10 min), clarified supernatants were removed and immediately flash-frozen with N_2_ and then freeze-dried for subsequent chemical characterisation. The solid pretreated biomasses were washed twice with deionised water, dried at 60°C, and stored for subsequent analyses and alkali pretreatment. For neutral sugars and lignin determinations, AIR samples were produced from this WRF-treated biomass (as described above).

### Mild Alkali Pretreatment

A portion of the non-WRF treated (NF) and WRF-treated solid fractions (approximately 250 mg, dry weight) were subjected to a mild alkali treatment with 2.5 mL 0.1 M NaOH for 24 h at 150 rpm shaking at 21°C. This step was performed with the aim of achieving biomass saponification, and to determine if combined WRF and mild alkali (WRF-ALK) pretreatments would act synergistically on the biomass, break ester-linkages, and release potentially valuable molecules. After the pretreatment, the samples were centrifuged (2000 × *g* for 10 min) and aliquots of the supernatants were flash-frozen with N_2_, and then freeze-dried for subsequent chemical characterisation. The pretreated solids were washed 3 times in 5 mL of 0.025M potassium acetate buffer (pH = 5.6) and twice with deionised water, dried at 60°C, and stored for subsequent assays. For neutral sugars and lignin determinations, AIR samples were produced as described above from this alkali (ALK)-treated biomass. Furthermore, the alkali pretreatment was also employed on AIR samples prepared from non-pretreated samples, to assess the exclusive effect of 0.1M NaOH on structural compounds. These samples are subsequently referred to as AIK.

### Fourier-Transform Infrared Spectroscopy

Attenuated total reflectance Fourier transform mid-infrared (FTIR-ATR) spectroscopy was performed on all samples included in this study (AIR, WRF-treated and mild alkali-treated samples), as reported elsewhere (da Costa et al., 2020; Marques et al., 2020). Duplicate spectra were collected in the range 4000–400 cm^–1^ using a Bruker Optics Vertex 70 FTIR spectrometer purged by CO_2_-free dry air and equipped with a Brucker Platinum ATR single reflection diamond accessory. A Ge on KBr substrate beamsplitter and a liquid nitrogen-cooled wide band mercury cadmium telluride (MCT) detector were used. Spectra were averaged over 32 scans at a resolution of 4 cm^–1^, and the 3-term Blackman-Harris apodization function was applied. The Bruker Opus 8.1 software was also used to: (i) remove eventual H_2_O and CO_2_ contributions, and (ii) spectral smoothing using the Savitzky-Golay algorithm (window: 17 pt.). Absorbance spectra were converted to text files, imported into MatLab (v. R2014b; MathWorks, Natick, MA, United States) and averaged. Full spectra, or fingerprint region spectra (1800–800 cm^–1^), were vector normalised to unit length and the baseline was removed according to the automatic weighted least squares algorithm (polynomial order = 2) prior to statistical analysis, using the Eigenvector PLS Toolbox (v. 7.9; Eigenvector Research, Wenatchee, WA, United States).

### Saccharification

Non-pretreated, WRF-treated (30-day incubation with *G*. *lucidum*, *T*. *versicolor* and *P*. *ostreatus*) and mild alkali-treated biomass samples were included in a saccharification assay, with four technical replicates for each sample, using an automatic platform as previously described by Gomez et al. (2010). Briefly, enzymatic hydrolysis was achieved using Cellic CTec2 (Novozymes, Bagsvaerd, Denmark) in a Na-Acetate buffer (25 mM; pH = 4.5) at 50°C. Cocktails were prepared so that cellulase loadings were 8 filter paper units (FPU) per g of biomass in the Na-Acetate buffer. Saccharification was measured after 8 h by colorimetric detection of reducing sugar equivalents as described by Whitehead et al. (2012).

### Neutral Monosaccharides

Acid hydrolysis and neutral monosaccharide determinations were performed as previously described (da Costa et al., 2017), on non-pretreated, WRF-treated (30-day incubation with *P*. *ostreatus*) and mild alkali-treated AIR samples. Briefly, 10 mg of each sample was weighed into 10 mL Pyrex glass tubes and 100 μL H_2_SO_4_ (72% w/w) was added. Sealed tubes were left at 30°C for 1 h. Samples were diluted to 4% H_2_SO_4_ (w/w) and autoclaved at 121°C for 1 h. Once at room temperature, hydrolysates were neutralised using CaCO_3_, and the tubes were centrifuged (2000 × *g* for 10 min) to obtain a clear supernatant. Carbohydrate separation and detection was achieved using high-performance anion exchange chromatography with pulsed amperometric detection (HPAEC-PAD). The ICS-5000 ion chromatography system (Dionex, Sunnyvale, CA, United States) was operated at 45°C using a CarboPac SA10 column with a CarboPac SA10G guard column. An eluent generator prepared 0.001 M KOH for 14 min isocratic elution at 1.5 mL min^–1^. Calibration standards were used for monosaccharide identification and quantitation.

### Lignin Measurement

Acetyl bromide soluble lignin percentages were determined in duplicate for non-pretreated, WRF-treated (30-day incubation with *P*. *ostreatus*) and mild alkali-treated AIR samples, as previously reported (da Costa et al., 2014). To approximately 10 mg of each sample, 500 μL of freshly prepared 25% (v/v) acetyl bromide solution in glacial acetic acid was added, the tubes were capped and left at 50°C for a total of 3h. Following lignin solubilisation, the tubes were cooled, and their contents were diluted by the addition of 2000 μL of 2 M NaOH. A further addition of 350 μL of 0.5 M hydroxylamine hydrochloride to each tube ensured the decomposition of polybromide ions (Monties, 1989). Final volumes were adjusted to 10 mL with glacial acetic acid and centrifuged (2000 × *g* for 10 min) to produce particulate-free supernatants. From there, 200 μL of each sample was transferred to UV-transparent 96-well plates (UV-Star, Greiner Bio-One). Absorbance at 280 nm was measured with a plate reader (Perkin Elmer, Multimode Plate Reader 2300 EnSpire). Blank negative controls were included and their absorbance at 280 nm was set as absorbance baseline. Lignin dry weight percentages were calculated as follows: *ABSL*% = (*A*_280_/(*SAC* × *PL*)) × (*V*_*R*_/*W*_*S*_) × 100%; where *ABSL*% is the acetyl bromide-soluble lignin percentage content; *A*_280_ is the absorption reading at 280 nm; *PL* is the pathlength determined for the 96-well microplates with a volume of 200 μL per well used during the analysis (0.556 cm); *V*_*R*_ is the reaction volume (litres); *W*_*S*_ is the sample weight (g); and *SAC* is the a specific absorption coefficient of 17.78 g^–1^ L cm^–1^, as reported for purified HCl-dioxane lignin from poalean samples (Lygin et al., 2011).

### Characterisation of Pretreatment Liquid Fractions

The freeze-dried pretreatment liquid fractions from WRF-treated (30-day incubation with *P*. *ostreatus*), mild alkali-treated, and non-pretreated samples (non-inoculated negative controls with deionised water) were reconstituted in 100% methanol. These were then kept at −20°C for 24 h and then centrifuged (14,000 × *g* for 5 min). The resulting supernatants consisted of clarified methanolic extracts, containing phenols of interest, but free of most sugars and other water-soluble compounds. Samples were analysed by reverse-phase HPLC equipped with a photodiode array detector and coupled with an electrospray ionisation tandem mass spectrometer (HPLC-PDA-ESI-MS^*n*^) on a Thermo Finnigan system (Thermo Electron Corp., Waltham, MA, United States), as described elsewhere (Bhatia et al., 2020). Separation of compounds was carried out on a Waters C18 Nova-Pak column (3.9 × 150 mm, particle size 4 μm) at 30°C with a flow rate of 1 mL/min and injection volume of 10 μL. The mobile phase consisted of water with 0.1% formic and acid (A) and methanol with 1% formic acid (B) with B increasing from 5 to 65% in 30min. Eluting compounds were detected with a Finnigan PDA Plus detector between 240 and 400 nm and a Finnigan LTQ linear ion trap with an ESI source. MS parameters were as follows: sheath gas flow 30, auxiliary gas flow 15 and sweep gas zero (arbitrary units), spray voltage −4.0 kV in negative and 4.8 kV in positive ionisation mode, capillary temperature 320°C, capillary voltage −1.0 and 45 V, respectively, tube lens voltage −68 and 110 V, respectively, and normalised collision energy (CE) typically 35%. Data were acquired and processed using Thermo Scientific^TM^ Xcalibur^TM^ software. Potential compounds of interest were characterised and/or tentatively identified by their UV and MS spectra or identified by direct comparison with authentic standards, or with fragmentation patterns reported elsewhere (Simirgiotis et al., 2013; Ostrowski et al., 2016). Approximate quantification of compounds was carried out by comparison of the area under the curve for selected *m/z* chromatograms in negative mode.

### Statistical Analysis

All univariate descriptive statistics, analyses of variance and Tukey’s range tests were performed using Statistica (v. 8.0; StatSoft, Tulsa, Oklahoma). For the *t*-tests on spectral data to unveil the underlying chemometric relationships between FTIR-ATR spectra, an R-based data analysis platform was used (Chong et al., 2018).

## Results

### Grass Cell Wall Compositional Characterisation

**FIGURE 2:**
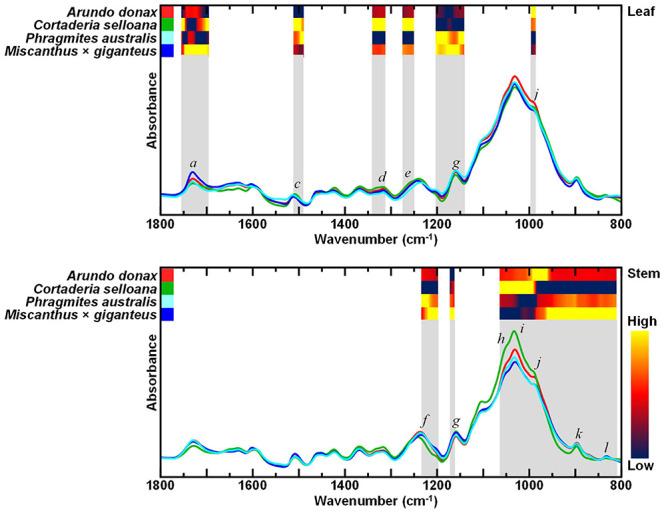
Attenuated total reflectance Fourier transform mid-infrared (FTIR-ATR) spectra of alcohol insoluble residue (AIR) of leaf and stem biomass from *Arundo donax*, *Cortaderia selloana*, *Phragmites australis* and *Miscanthus* × *giganteus*. Grey shading highlights the most significantly different regions of the spectra, based on ANOVA (*p* ≤ 0.00001), while the heatmaps highlight the relative absorption intensities according to the grass species. Spectral bands of interest are marked from *a* – *l*, see text and Table 1 for more information.

Alcohol insoluble residues (AIR) were prepared for the compositional characterisation of cell wall biomass from *A*. *donax*, *C*. *selloana*, *P*. *australis*, and *M*. × *giganteus*, and examined by FTIR-ATR spectroscopy. Resulting data underwent analysis of variance (ANOVA), to find the most significantly different spectral regions between the different species. A low *p*-value threshold (*p* ≤ 0.00001) was chosen to expose the most significantly different wavenumbers. Heatmaps in the most distinct spectral regions highlight the relative chemometric differences between the different spontaneous grasses, and in relation to *M*. × *giganteus*, a prospective dedicated lignocellulosic crop for biorefining applications (Figure 2).

For leaf cell wall biomass, six contiguous intervals were the most significantly different spectral regions (cm^–1^): 1754 – 1695, 1510 – 1489, 1339 – 1311, 1281 – 1256, 1201 – 1140, 995 – 985. For stem, three intervals showed the most significant differences (cm^–1^): 1232 – 1197, 1171 – 1161, 1063 – 809. In the FTIR spectra of leaves, spectral regions *e* (1275 – 1256 cm^–1^) and *d* (1322 – 1310 cm^–1^) have been assigned to lignin structural features (Table 1). Bands *a* (1736 – 1730 cm^–1^), and *g* (1170 – 1160 cm^–1^) have been attributed to C = O stretching in acetyl-xylans, and to O-C-O asymmetric stretching in glycosidic links, respectively (Table 1). Lastly, spectral region *j* (993 – 985 cm^–1^) includes wavenumbers associated with vibrations in cellulose.

**TABLE 1:** Assignment of relevant FTIR-ATR absorption bands characteristic of cell wall biomass from poalean species.

**Region**	**Absorption (cm^–1^)**	**References**	**Assignment**	**Cell wall feature**
*a*	1736 – 1730	1735 (Gwon et al., 2010) 1730 (Sills and Gossett, 2012) 1735 (Bekiaris et al., 2015)	C = O vibrations	Xylan
*b*	1625 – 1635	1630 (Chatjigakis et al., 1998) 1630 (Manrique and Lajolo, 2002) 1630 (Gannasin et al., 2012)	COO^–^ vibrations	Non-esterified carboxyl groups
*c*	1515 – 1505	1515 (McCann et al., 2001) 1510 (Bekiaris et al., 2015) 1513 (Zhang et al., 2017)	Aromatic ring vibration	Lignin and other phenols
*d*	1322 – 1310	1315 (Pandey, 1999) 1317 (Chen et al., 2010) 1320 (Bekiaris et al., 2015)	Syringyl monomer vibration	Lignin
*e*	1275 – 1256	1270 (Tejado et al., 2007) 1270 (Tan et al., 2009) 1268 (Jiang et al., 2015)	Guaiacyl monomer vibration	Lignin
*f*	1240 – 1235	1240 (Marchessault, 1962) 1235 (Harrington et al., 1964) 1240 (Bekiaris et al., 2015)	C-O vibrations of acetyl	Xylan
*g*	1170 – 1160	1160 (Kačuráková et al., 2000) 1161 (Abidi et al., 2014) 1160 (Szymanska-Chargot and Zdunek, 2013) 1160 (Bekiaris et al., 2015)	O-C-O asymmetric stretching (glycosidic link) all residues	Polysaccharides
*h*	1060 – 1055	1055 (Harrington et al., 1964) 1060 (Wilson et al., 2000) 1060 (Schulz and Baranska, 2007)	C-O, C-C and O-C-H vibration	Cellulose
*i*	1035 – 1030	1035 (Wilson et al., 2000) 1035 (Schulz and Baranska, 2007)	C-O, C-C and C-C-O stretching	Cellulose
*j*	993 – 985	990 (Pastorova et al., 1994) 993 (Gwon et al., 2010) 990 (Marry et al., 2000) 993 (Oh et al., 2005)	C-O stretching	Cellulose
*k*	898 – 890	893 (Oh et al., 2005) 898 (Ciolacu et al., 2011) 898 (Bekiaris et al., 2015)	C-O-C asymmetric stretching	Cellulose (amorphous)
*l*	840 – 830	835 (Harrington et al., 1964) 834 (Faix, 1991) 838 (Zhang et al., 2017)	C-H out-of-plane bending in syringyl and *p*-hydroxy-phenyl monomers	Lignin

For stems, the spectral regions showing the greatest significant differences between the species also include bands associated with cellulose. Specifically, bands *h* (1060 – 1055 cm^–1^), *i* (1035 – 1030 cm^–1^) and *k* (898 – 890 cm^–1^) which is associated with amorphous cellulose structures (Table 1). For the spectra of stem biomass, the most significantly different regions also included band *g* (1170 – 1160 cm^–1^), which has been ascribed to vibrations in glycosidic links (Table 1), and to vibrations in non-cellulosic cell wall components. The latter included band *f* (1240 – 1235 cm^–1^), associated with xylans, and band *l* (840 – 830 cm^–1^), assigned to lignin structures (Table 1).

Together these results suggest that despite the compositional similarity between the biomasses of these different poalean species, key significantly differences may have an impact in their biorefining performance.

### Effect of Mild Alkali and White-Rot Fungi Pretreatments on Grass Biomass Composition

**FIGURE 3:**
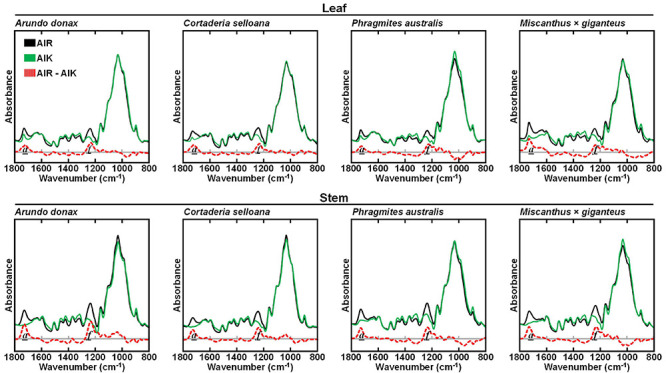
Attenuated total reflectance Fourier transform mid-infrared (FTIR-ATR) spectra of untreated alcohol insoluble residue (AIR) and pretreated with 0.1 M NaOH for 24 h at 21°C (AIK), for leaf and stem biomass from *Arundo donax*, *Cortaderia selloana*, *Phragmites australis* and *Miscanthus* × *giganteus*. The difference between AIR and AIK biomasses are presented by a red dashed line. Spectral bands of interest are marked as: *a* (1736 – 1730 cm^–1^) and *f* (1240 – 1235 cm^–1^).

To assess the effect of a mild alkali pretreatment on the cell wall from *A*. *donax*, *C*. *selloana*, *P*. *australis*, and *M*. × *giganteus*, AIR samples were treated with 0.1 M NaOH for 24 h at 21°C (AIK). FTIR-ATR was subsequently employed to assess the main compositional changes effected by the alkali. For leaf and stem of the four examined grasses, the most marked differences between the spectra of AIR and AIK samples were observed in the *a* (1736 – 1730 cm^–1^) and *f* (1240 – 1235 cm^–1^) spectral regions (Figure 3). Band *a*, centred at 1735 cm^–1^ and assigned to C = O stretching in xylans (Table 1), showed reduced intensity in pretreated biomass. Concomitantly, the intensity of band *f*, associated to C-O vibrations of acetyl, is also reduced.

**FIGURE 4:**
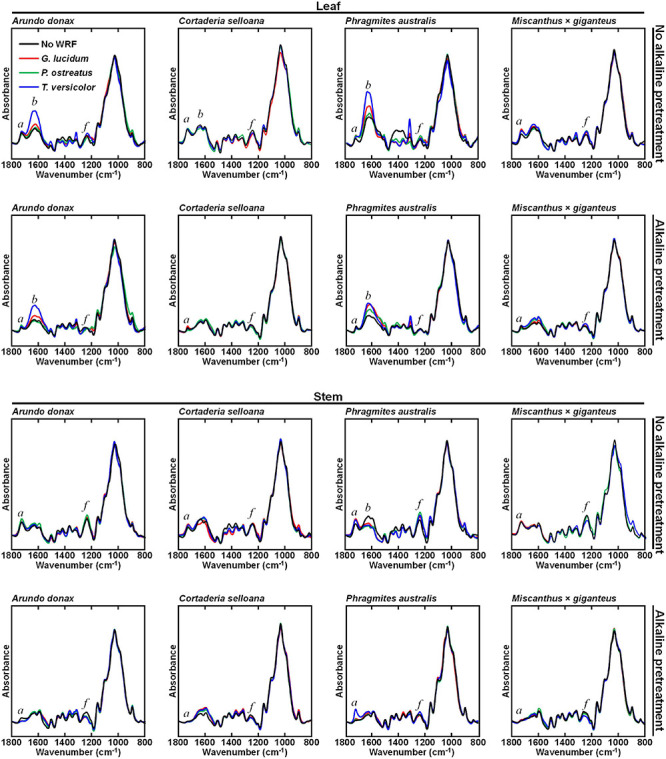
Attenuated total reflectance Fourier transform mid-infrared (FTIR-ATR) spectra of milled but not organic solvent-washed biomass samples from *Arundo donax*, *Cortaderia selloana*, *Phragmites australis* and *Miscanthus* × *giganteus*, treated with white-rot fungi (WRF; 30 days) and/or in combination with 0.1 M NaOH (24 h at 21°C) alkali pretreatment. Three fungal species were used: *Ganoderma lucidum*, *Pleurotus ostreatus* and *Trametes versicolor*. Control samples consist of biomass incubated only with water, without WRF. Spectral bands of interest are marked as: *a* (1736 – 1730 cm^–1^), *b* (1625 – 1635 cm^–1^) and *f* (1240 – 1235 cm^–1^).

Another approach to biomass fractionation, which may lead to an increase in lignocellulosic biodegradability and the release of valuable molecules, is the pretreatment of biomass using white-rot fungi (WRF). The WRF-treated samples were subjected to FTIR-ATR examination to reveal the main effects of the pretreatments on biomass composition (Figure 4). Interestingly, the effect of a given WRF species varies between different biomass species. Similarly, the same biomass is differently affected by each WRF species. Leaf biomass composition does not appear to be greatly affected in *M*. × *giganteus* and *C*. *selloana* when only WRF treatments are employed. In stems, biomass treated with WRF alone generally does not appear to be modified in relation to the non-WRF treated (NF) control samples, except in *P*. *australis* stems, where non-pretreated samples showed higher intensities for the *b* (1625 – 1635 cm^–1^).

By contrast, when the combined WRF-ALK treatment is employed, similar modifications were seen to when AIR and AIK samples were compared (Figure 3). As previously discussed, modifications induced by the mild alkali pretreatment are primarily observed at *a* (1736 – 1730 cm^–1^) and *f* (1240 – 1235 cm^–1^) spectral regions (Figure 4), which have been credited to a decrease in acetylation of xylans. In leaf samples treated with WRF and with the combined WRF-ALK pretreatment, the grass species with greater compositional modifications are *A*. *donax* and *P*. *australis*. In stems, it is noteworthy that for *P*. *australis* the intensity of band *a* is not reduced in biomass treated with *T. versicolor* and 0.1 M NaOH, as observed with the remaining biomasses after being treated with alkali. For both leaf and stem, the most striking spectral modification is in the band centred at 1630 cm^–1^ (*b*; Table 1), which has been assigned to non-esterified carboxyl groups in polysaccharides.

### Saccharification Yields of Grass Biomass

**FIGURE 5:**
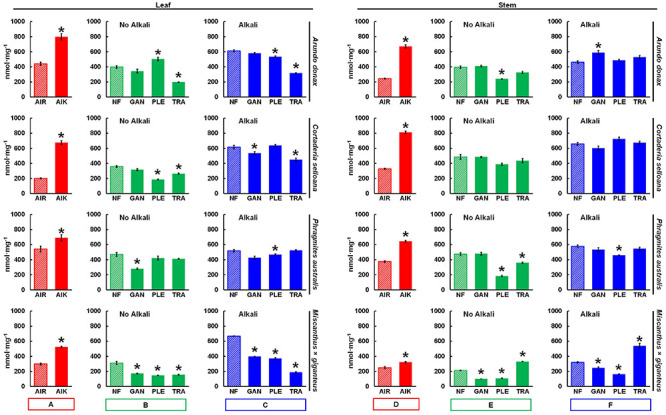
Saccharification of leaf and stem biomass from four poalean species measured by a high-throughput saccharification assay: *Arundo donax*, *Cortaderia selloana*, *Phragmites australis* and *Miscanthus* × *giganteus*. Mean nmol of reducing sugar released per mg of biomass material (nmol mg^–1^) after 8 h incubation in a hydrolytic enzyme mixture. Red bars **(A,D)** refer to non-pretreated alcohol insoluble residue samples, without (AIR) or with (AIK) a 0.1 M NaOH mild alkali pretreatment. Green bars **(B,E)** refer to samples treated only with one of the following white rot fungi (WRF): *Ganoderma lucidum*, GAN; *Pleurotus ostreatus*, PLE; *Trametes versicolor*, TRA. Blue bars **(C,F)** designate treatments where the 0.1 M NaOH treatment was employed subsequently to the WRF pretreatment. Pairwise *t*-tests were performed between each treatment and non-pretreated control samples (AIR; or NF, no fungi; striped bars) to evaluate the impact of the pretreatments. The treatments which are significantly different from the controls are marked with a “*” (*p* ≤ 0.05). Error bars represent the standard error of the sample replicates. For the saccharification values see Supplementary Table 1.

To compare biorefining potentials between the different poalean species, and to investigate the effect of the applied pretreatments, enzymatic saccharification assays were performed on all samples. AIR prepared from *P*. *australis* biomass showed the highest saccharification yields in leaves and in stems, respectively (Figure 5): 541.8 and 375.2 nmol mg^–1^ after 8h incubation (Supplementary Table 1). The AIR samples were also treated with a mild alkali pretreatment (AIK) 0.1 M NaOH (24 h at 21°C). In these samples, *A*. *donax* showed the highest saccharification yield among leaf samples (799.2 nmol mg^–1^), whereas for stem the highest yield was seen with *Cortaderia selloana* (812.1 nmol mg^–1^). By contrast, *M*. × *giganteus* typically showed comparatively low saccharification yields. Saccharification yields were also typically higher from leaves than stems, except for *C*. *selloana*, which showed higher sugar yields from stem AIR and AIK samples (Figures 5A,D; red bars).

For each of the applied pretreatments, the percentage of recovered solids was calculated (Supplementary Table 2) for each grass species and organ. From WRF-treated leaf and stem samples, typically 97% of the biomass is recovered after a 30-day incubation. When the 0.1M NaOH mild alkali pretreatment alone is employed, 97% of leaf and 96% of stem biomass is recovered. As for combined WRF-ALK pretreatments, recovered percentages drop slightly to 95% from leaves, and 94% from stems.

Biomass treated with WRF showed increased saccharification yields in certain conditions when compared with NF controls (Figures 5B,E; green bars). The highest saccharification yield from samples treated with WRF alone was seen using *P*. *ostreatus* on *A*. *donax* leaf biomass (503.7 nmol mg^–1^; Figure 5), 26.4% higher than NF controls (*p* ≤ 0.05). While stem biomass treated with *T*. *versicolor* showed a lower saccharification yield (331.1 nmol mg^–1^), these WRF-treated samples yielded 55.2% more sugars than *M*. × *giganteus* samples (213.2 nmol mg^–1^; *p* ≤ 0.05). Stem biomass from *A*. *donax* and *P*. *australis* treated with *G*. *lucidum* also showed marginally higher saccharification yields than the NF controls, although, for most samples treated only with WRF, the saccharification yields were lower than the NF controls.

A mild alkali pretreatment (0.1 M NaOH; 24 h; 21°C) was also employed in sequence with the WRF pretreatment (Figures 5C,F; blue bars) to extract compounds which may be of interest from an application perspective. This alkaline treatment substantially increased saccharification yields. The highest, significantly different (*p* ≤ 0.05) saccharification yield in relation to the controls was seen with *A*. *donax* stems treated with *G*. *lucidum* and 0.1M NaOH (589.4 nmol mg^–1^). However, the overall highest yield seen in samples treated with this combined WRF-ALK treatment was with *C*. *selloana* stems treated with *P*. *ostreatus* (725.2 nmol mg^–1^).

For leaves pretreated with WRF alone, the highest saccharification yield was seen with *P*. *ostreatus*-treated *A*. *donax* (503.7 nmol mg^–1^), whereas with the combined WRF-ALK approach, the highest yield was also observed with *P*. *ostreatus*-treated biomass, but in *C*. *selloana* samples (637.9 nmol mg^–1^; Supplementary Table 1 and Figures 5B,C). In stems treated with the WRF-ALK combination, the highest yield was once again with *P*. *ostreatus* on *C*. *selloana* biomass (725.2 nmol mg^–1^; Supplementary Table 1 and Figure 5F), although when only the WRF pretreatment was employed, the highest yield was obtained with *G*. *lucidum*. Given that three out of the four highest saccharification yields were obtained from biomasses treated with *P*. *ostreatus*, subsequent cell wall and pretreatment liquid fraction compositional analyses were performed only for samples treated with this ligninolytic fungal species.

### Grass Cell Wall Neutral Sugars and Lignin Composition

**FIGURE 6:**
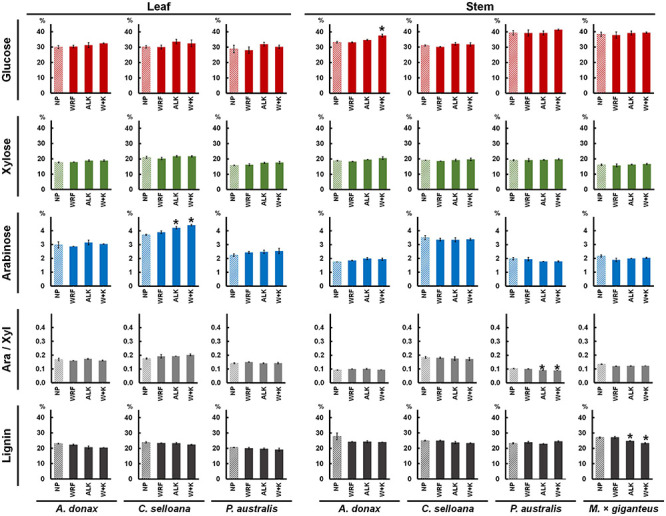
Mean percentage (%) composition of alcohol insoluble residues (AIR) prepared from previously pretreated biomass from *Arundo donax*, *Cortaderia selloana*, *Phragmites australis* and *Miscanthus* × *giganteus* (no results are shown for *M.* × *giganteus* leaves due to sample losses). Pretreatment acronyms: NP, non-pretreated control samples (incubated only with water; striped bars); WRF, samples pretreated only with *Pleurotus ostreatus*; ALK, samples pretreated only with 0.1 M NaOH for 24 h at 21°C; W + K, samples pretreated with *P*. *ostreatus* followed by 0.1 M NaOH for 24 h at 21°C. Ara/Xyl is the ratio of arabinose to xylose determined in the biomasses. Pairwise *t*-tests were performed between each treatment and control (NP) samples to evaluate the impact of the pretreatments in relation to NP samples for each variable, the treatments which are significantly different from the NP controls are marked with a “*” (*p* ≤ 0.05). Error bars represent the standard error of the sample replicates.

Main cell wall neutral sugars and lignin contents were determined for leaf and stem samples from the grass species examined in this study. In *A*. *donax*, *C*. *selloana* and *P*. *australis*, average leaf cell wall composition was 29.7% glucose, 18.2% xylose and 3.0% arabinose (Figure 6 and Table 2) of AIR dry weight (DW) for non-pretreated control samples (only incubated with water). Glucose content did not vary significantly (*p* > 0.05) much between species, but xylose and arabinose were typically higher in *C*. *selloana* leaves (21.1% and 3.7% DW respectively). In stems, the corresponding values were: 34.5% glucose, 19.1% xylose and 2.4% arabinose DW, respectively, with highest glucose and xylose being observed in *P*. *australis* (39.4% and 19.2% DW respectively), while for highest arabinose content was observed in *C*. *selloana* (3.5%).

**TABLE 2:** Mean percentage (%) composition of alcohol insoluble residues (AIR) prepared from previously pretreated biomass from *Arundo donax*, *Cortaderia selloana*, *Phragmites australis* and *Miscanthus* × *giganteus*.

	**Leaf**					
	**Glucose**	**Xylose**	**Arabinose**	**Arabinose to Xylose ratio**	**Lignin**	
**NP**	30.0 ± 1.0	17.7 ± 0.3	3.0 ± 0.2	0.17 ± 0.01	22.9 ± 0.4	***A. donax***
**WRF**	30.3 ± 0.8	17.9 ± 0.1	2.9 ± < 0.1	0.16 ± < 0.01	22.2 ± 0.5	
**ALK**	31.2 ± 1.7	18.9 ± 0.5	3.2 ± 0.2	0.17 ± < 0.01	20.6 ± 0.9	
**WRF + ALK**	32.5 ± 0.3	18.9 ± 0.5	3.0 ± < 0.1	0.16 ± < 0.01	20.3 ± 0.1	
**NP**	30.2 ± 0.9	21.1 ± 0.8	3.7 ± < 0.1	0.18 ± < 0.01	23.8 ± 0.5	***C. selloana***
**WRF**	30.1 ± 1.3	20.3 ± 0.8	3.9 ± 0.1	0.19 ± 0.01	23.4 ± 0.2	
**ALK**	33.6 ± 1.5	21.8 ± 0.5	4.2 ± 0.1	0.19 ± < 0.01	23.3 ± 0.6	
**WRF + ALK**	32.5 ± 2.2	21.8 ± 0.5	4.4 ± 0.1	0.20 ± 0.01	22.4 ± 0.3	
**NP**	28.8 ± 2.4	15.8 ± 0.2	2.2 ± 0.1	0.14 ± < 0.01	20.5 ± 0.1	***P. australis***
**WRF**	28.0 ± 2.2	16.2 ± 0.7	2.4 ± 0.1	0.15 ± < 0.01	20.1 ± 0.6	
**ALK**	31.9 ± 1.3	17.5 ± 0.4	2.5 ± 0.1	0.14 ± < 0.01	19.6 ± 0.6	
**WRF + ALK**	30.2 ± 1.1	17.7 ± 0.7	2.5 ± 0.2	0.14 ± 0.01	19.1 ± 1.0	
**NP**	33.2 ± 0.6	18.8 ± 0.3	1.8 ± < 0.1	0.09 ± < 0.01	27.8 ± 2.2	***A. donax***
**WRF**	33.2 ± 0.3	18.4 ± 0.2	1.9 ± < 0.1	0.10 ± < 0.01	24.3 ± 0.1	
**ALK**	34.7 ± 0.3	19.6 ± 0.1	2.0 ± 0.1	0.10 ± < 0.01	24.3 ± 0.7	
**WRF + ALK**	37.6 ± 1.0	20.6 ± 0.9	1.9 ± 0.1	0.10 ± < 0.01	24.1 ± < 0.1	
**NP**	31.0 ± 0.5	19.1 ± 0.1	3.5 ± 0.2	0.18 ± 0.01	24.9 ± 0.3	***C. selloana***
**WRF**	30.1 ± 0.2	18.6 ± < 0.1	3.4 ± 0.1	0.18 ± 0.01	25.0 ± 0.3	
**ALK**	32.1 ± 0.9	19.2 ± 0.6	3.3 ± 0.1	0.18 ± 0.01	23.7 ± 0.7	
**WRF + ALK**	31.7 ± 1.2	19.7 ± 0.8	3.4 ± 0.1	0.17 ± 0.01	23.3 ± 0.3	
**NP**	39.4 ± 1.4	19.2 ± 0.5	2.0 ± 0.1	0.10 ± < 0.01	23.3 ± 0.4	***P. australis***
**WRF**	39.2 ± 2.0	19.3 ± 1.0	2.0 ± 0.1	0.10 ± < 0.01	24.0 ± 0.7	
**ALK**	39.3 ± 1.3	19.5 ± 0.2	1.8 ± < 0.1	0.09 ± < 0.01	22.9 ± 0.3	
**WRF + ALK**	41.4 ± 0.4	19.8 ± 0.4	1.8 ± < 0.1	0.09 ± < 0.01	24.5 ± 0.4	
**NP**	38.3 ± 2.9	16.2 ± 0.4	2.2 ± < 0.1	0.13 ± < 0.01	27.0 ± < 0.1	***M.* × *giganteus***
**WRF**	37.8 ± 1.0	15.7 ± 0.2	1.9 ± < 0.1	0.12 ± < 0.01	27.1 ± 0.3	
**ALK**	39.1 ± 0.3	16.3 ± 0.8	2.0 ± 0.1	0.12 ± < 0.01	24.8 ± 0.5	
**WRF + ALK**	39.4 ± 0.3	16.7 ± 0.3	2.0 ± < 0.1	0.12 ± < 0.01	23.4 ± 0.3	

*Pretreatment acronyms: NP, non-pretreated control samples; WRF, samples pretreated only with *Pleurotus ostreatus*; ALK, samples pretreated only with 0.1 M NaOH for 24 h at 21°C; WRF + ALK, samples pretreated with *P*. *ostreatus* followed by 0.1 M NaOH for 24h at 21°C. Values are the mean ± standard error of the sample replicates.*

In biomasses treated with WRF, 0.1M NaOH mild alkali or a combination of both, in most cases, neutral sugar composition did not change significantly in relation to the non-pretreated samples (*p* > 0.05). The only exceptions were glucose in *A*. *donax* leaf biomass treated with the combined WRF-ALK pretreatment, where the values were slightly higher than in non-pretreated biomass (37.6% versus 33.2% DW; *p* ≤ 0.05). Similarly, for arabinose, the values were statistically higher than the non-pretreated samples for *C*. *selloana* leaves treated with mild alkali or the combined WRF-ALK pretreatments (up from 3.7% to 4.2% and 4.4% DW, respectively; *p* ≤ 0.05). These slight increases in neutral sugars in alkali-pretreated samples are likely related to the pretreatment effect, which improved acid hydrolysis efficiency. An estimation of arabinoxylan (AX) degree of ramification was also achieved by calculating arabinose to xylose ratios (Ara/Xyl). In non-pretreated samples, Ara/Xyl ratios averaged to 0.16 in leaves, and 0.13 in stems (Figure 6). For both leaf and stem, the highest values were seen in *C*. *selloana*, with an Ara/Xyl ratio of 0.18 (Table 2).

For lignin in non-pretreated cell wall biomass (Figure 6), average values across the spontaneous grasses were 22.4% in leaves (23.8% in *C*. *selloana*, 22.9% in *A*. *donax* and 20.5% DW in *P*. *australis*; Table 2) and 25.3% in stems (27.8% in *A*. *donax*, 24.9% in *C*. *selloana*, and 23.3% DW in *P*. *australis*). In pretreated samples, there was a decrease in lignin content, albeit only significantly lower in relation to non-pretreated samples in *M*. × *giganteus* treated with 0.1M NaOH and with the combined WRF-ALK treatments (a decrease from 27.0% down to 24.8% and 23.4%, respectively; *p* ≤ 0.05; Figure 6).

### Analysis of Pretreatment Liquid Fractions

The liquid fractions derived from the fungal and mild alkali pretreatments, including the NF control samples (incubated only with water) were analysed by liquid chromatography with detection by photodiode array and tandem mass spectrometry (HPLC-PDA-ESI-MS^*n*^). Several negative ions, corresponding to lignin-derived compounds were detected in the pretreatment liquid fractions. These included *p*-coumaric acid (*m/z* 163), hydroxybenzoic acid (*m/z* 121) and ferulic acid (*m/z* 193). Other cell wall related compounds that were tentatively identified include diferulic acid isomers (*m/z* 385) and diferulic acid cyclobutane isomers (*m/z* 387). Other prevalent ions included *m/z* 563 and *m/z* 389, with the former producing a UV spectrum and MS^2^ fragment ions consistent with apigenin-*C*-hexoside-*C*-pentoside (AHP). Multiple forms of *m/z* 389 ions (M_*r*_ 390) were detected in samples.

Both alkali pretreatment and tissue type showed a relationship with pretreatment liquid fraction composition. Coumaric acid and hydroxybenzaldehyde were observed in all samples following incubation with alkali, irrespective of tissue type or fungal pretreatment, while levels were negligible with other treatments (see Table 3). Ferulic acid is relatively abundant in grass cell wall tissues; however, here the amounts of this acid were relatively low or even absent, apart from leaf tissue pretreatment liquid fractions obtained from *A. donax* and *P. australis.* This pattern was also reflected in the content of diferulic acid and diferulic acid cylobutane. These compounds were observed in other samples but did not show an obvious relationship with treatment type or species. Certain compounds did show greater abundance following fungal pretreatments, however these were not consistent between species. AHP levels were raised in *P. australis* treated with *P. ostreatus*. However, this was not evident with other species. A compound with *m/z* 436 was also observed in *M*. × *giganteus* samples following fungal pretreatment, but again this observation was only specific to this species. AHP (apigenin-C-hexoside-C-pentoside) levels increased in *P*. *australis* treated with *P*. *ostreatus*, however this was not evident with other species. This compound was the only flavonoid observed in these pretreatment liquid fractions with the exception of the related compound apigenin-C-pentoside-C-pentoside in *A*. *donax* and tricin in *C*. *selloana* pretreatment liquid fraction.

**TABLE 3:** Abundance of compounds observed in pretreatment liquid fractions obtained from leaf and stem plant material.

	**Leaf**				**Stem**				
	**NP**	**WRF**	**ALK**	**W + K**	**NP**	**WRF**	**ALK**	**W + K**	
Coumaric acid	ND	ND	+ +	+ + + +	ND	ND	+ +	+ + +	***Arundo donax***
Hydroxybenzoic acid	ND	ND	Trace	+ + +	ND	ND	+	+ + +	
Ferulic acid	+	+ +	+	+	ND	ND	+	ND	
Diferulic acid isomers	+ + +	+ + +	ND	+ +	+ + +	ND	ND	ND	
Ferulic acid cyclobutane isomers	+ + + +	+ + +	ND	+ +	+ + +	Trace	ND	Trace	
Apigenin-C-pentoside-C-pentoside	ND	+	ND	+	+	ND	ND	+	
Apigenin-C-hexoside-C-pentoside	+	+	+	+ +	+ +++	+	+ +	+ +	
Coumaric acid	ND	ND	+ + +	+ + + +	ND	ND	+ +	+ + + +	***Cortaderia selloana***
Hydroxybenzoic acid	ND	ND	+ +	+ + +	ND	ND	+ + +	+ + +	
Ferulic acid	Trace	ND	ND	ND	ND	ND	ND	ND	
Diferulic acid isomers	+ +	+ + +	+ + +	+ +	+	+ +	+	+ +	
Ferulic acid cyclobutane isomers	+ +	+ + +	+ +	+	+	+	+	+	
Apigenin-C-pentoside-C-pentoside	ND	ND	ND	ND	ND	ND	ND	ND	
Apigenin-C-hexoside-C-pentoside	+ +	+	+ +	++	+ ++	++ + +	+ +++	++ + +	
Compound M_*r*_ 436	ND	ND	ND	ND	ND	ND	ND	ND	
Coumaric acid	ND	ND	+ + + +	+ +	ND	Trace	+ + + +	+ + + +	***Phragmites australis***
Hydroxybenzoic acid	ND	ND	+	+	Trace	ND	+ + +	+ + +	
Ferulic acid	+ + +	+ + +	+	+	Trace	Trace	ND	ND	
Diferulic acid isomers	+ + + +	+ + +	+ +	+ +	+ +	+ +	Trace	+	
Ferulic acid cyclobutane isomers	+ + + +	+ +	+ +	+ +	Trace	+	+	+	
Apigenin-C-pentoside-C-pentoside	ND	+ + + +	ND	+ + +	Trace	+ + +	+	+ +	
Apigenin-C-hexoside-C-pentoside	ND	+ + + +	ND	+ + +	ND	+ + + +	ND	+ + + +	
Compound M_*r*_ 436	ND	ND	ND	ND	ND	ND	ND	ND	
Coumaric acid					ND	ND	+ + + +	+ + + +	***Miscanthus* × *giganteus***
Hydroxybenzoic acid					ND	ND	+ + + +	+ + +	
Ferulic acid					Trace	Trace	ND	ND	
Diferulic acid isomers					ND	+	Trace	Trace	
Ferulic acid cyclobutane isomers					ND	+ +	+	+	
Apigenin-C-pentoside-C-pentoside					ND	ND	ND	ND	
Apigenin-C-hexoside-C-pentoside					ND	ND	ND	ND	
Compound M_*r*_ 436					ND	+ + + +	ND	+ + + +	

*The apigenin-*C*-hexoside-*C*-pentoside (AHP) compound schaftoside is commonly reported in grasses, however there are other flavonoids that are also relatively abundant in grasses. Furthermore, there are also reports of flavonoid biosynthesis in white rot fungi (Gąsecka et al., 2015). Pretreatments: NP, non-pretreated control samples (incubated only with water); WRF, samples pretreated with *Pleurotus ostreatus*; ALK, samples pretreated with 0.1 M NaOH for 24 h at 21°C; W + K, samples pretreated with *P*. *ostreatus* followed by 0.1 M NaOH for 24h at 21°C. The symbol “+” indicates levels detected, ND, not detected; Trace, very low levels detected.*

## Discussion

### Characterisation of the Biomass From Wild-Grown Spontaneous Grasses in Comparison to *Miscanthus × giganteus*

To determine the biorefining potential of spontaneous *A*. *donax*, *P*. *australis* and *C*. *selloana* from marginal lands in Portugal, their biomass composition was studied using a variety of analytical techniques. Furthermore, *Miscanthus* × *giganteus* (MXG), a potential energy crop was included in the study, for comparison with the wild biomass species. This crop has been proposed as a dedicated lignocellulosic crop in Europe (Lewandowski et al., 2000; Heaton et al., 2008), and has been extensively characterised from physiological, agronomical and potential application perspectives down to its cell wall composition and structure (de Souza et al., 2015; Clifton-Brown et al., 2016; Lewandowski et al., 2016; Van Der Weijde et al., 2016; da Costa et al., 2018; da Costa et al., 2019; Bhatia et al., 2021).

The contribution of leaf biomass to total biomass has not been determined in the present work. However, these percentages were previously determined in a range of *Miscanthus* genotypes (da Costa et al., 2014). Comparably to what was shown in that study, as the *bauplan* of *A*. *donax*, *C*. *selloana* and *P*. *australis* is similar to that of *Miscanthus* spp., leaf percentage contributions should also represent *ca*. 50% of total biomass in non-senesced plants (ranging between 40% – 60%). The separate analysis of leaf and stem, instead of pooled total above-ground biomass, as each of these types of biomass have very distinct properties in a biorefinery context.

Alcohol insoluble residues (AIR) were prepared from non-pretreated biomass samples from leaf and stem from the four grass species under study. From this material, neutral sugars, and acetyl bromide soluble lignin contents were determined. In all spontaneous species glucose and lignin content was higher in stems than in leaves, whereas for arabinose the amounts were higher in leaves. These values agree with previously reported data for grass species (Mann et al., 2009; Shen et al., 2009; Le Ngoc Huyen et al., 2010; da Costa et al., 2017). Xylose contents are higher in stems for *A*. *donax* and *P*. *australis*, which agree with the values reported elsewhere. However, for *C*. *selloana*, xylose contents are higher in leaves. No reports could be found in the literature about *Cortaderia* spp., however higher xylose percentages in stems would be expected for a grass species.

The cell wall molar ratio of arabinose to xylose can be used as indicator of the degree of arabinose substitution in arabinoxylans, which are the most abundant hemicellulose compounds in grass cell walls (Carpita, 1996). Arabinose to xylose ratios have been positively correlated with enzymatic saccharification efficiency of glucose (da Costa et al., 2019), and may be a good indicator of the potential applications of biomass crops. These ratios are typically highest in leaves and lowest in stems (Rancour et al., 2012), which agrees with the values observed for *A. donax* (0.17 in leaves and 0.09 in stems) and for *P. australis* (0.14 in leaves and 0.10 in stems). However, in *C*. *selloana* the arabinose to xylose ratio is the same in leaves and in stems (0.18).

The AIR from non-pretreated biomass from *M*. × *giganteus* stems contained 38.3% of glucose, 16.2% of xylose, 2.2% of arabinose and, 27.0% of lignin (Figure 6 and Table 2). These values are in accordance with previously reported determinations in 8 miscanthus genotypes harvested from the same location (da Costa et al., 2019). For each cell wall trait, a comparison can be made between these values and those of stem biomass from the spontaneous grasses under study. For glucose, the stems of *P. australis* contain higher amounts of glucose (39.4%) than *M*. × *giganteus*, whereas all three spontaneous species contain higher amounts of xylose (*A*. *donax*: 18.8%; *C*. *selloana*: 19.1%; *P*. *australis*: 19.2%), whereas lower amounts of lignin are found in *C*. *selloana* and in *P*. *australis* (24.9% and 23.3% respectively).

From the FTIR-ATR analysis it was observed that the spectra of AIR prepared from leaf biomass are mostly significantly different in regions ascribed to hemicelluloses, namely to acetyl-xylans (bands *a* 1736 – 1730 cm^–1^; *g* 1060 – 1055 cm^–1^; Table 1), to lignin (bands *c* 1322 – 1310 cm^–1^; *d* 1275 – 1256 cm^–1^ and *e* 1240 – 1235 cm^–1^), and to amorphous cellulose (band *j* 898 – 890 cm^–1^). With respect to xylan, bands *a* and *g* are highly positively and significantly correlated to each other (*r* > 0.8; *p* < 0.01). For the lignin-associated bands *c*, *d* and *e*, the decreasing band intensities across the grass species (*C*. *selloana* > *A*. *donax* > *P*. *australis*; Figure 2) coincide with the corresponding lignin determinations (Figure 6 and Table 2) in non-pretreated leaf biomass: *C*. *selloana* (23.8%), *A*. *donax* (22.9%) and *P*. *australis* (20.5%). Furthermore, spectral regions *e* and *d* are highly positive and significantly correlated to each other (*r* > 0.9; *p* < 0.01; Supplementary Figure 1). Finally, for band *j*, which has been assigned to amorphous cellulose, its decreasing intensity across the grass species (*A*. *donax* > *C*. *selloana* > *P*. *australis*; Figure 2) agrees with determined glucose percentages, as *C*. *selloana* and *A*. *donax* have the highest glucose values (30.2% and 30.0% respectively), and *P*. *australis* has the lowest (28.8%; Figure 6 and Table 2).

FTIR-ATR spectral bands showing the greatest significant differences in AIR from stems, for the different grass species (Figure 2) include: *h* (1060 – 1055 cm^–1^), *i* (1035 – 1030 cm^–1^) and *k* (898 – 890 cm^–1^). Although these bands have all been attributed to cellulose (Table 1), correlation analysis (Supplementary Figure 1) shows that while *h* and *i* band intensities are highly positively correlated to each other (*r* > 0.9; *p* < 0.01), they are both highly negatively correlated to band *k* (*r* < −0.9; *p* < 0.01), which has been associated with amorphous cellulose structures. The intensities of band *k* decrease across the grass species in the following order: *P*. *australis* > *A*. *donax* > *C*. *selloana*. This agrees with the order of their respective glucose contents (Figure 6 and Table 2): *P*. *australis* (39.4%), *A*. *donax* (33.2%) and *C*. *selloana* (31.0%). By contrast, the trend in the intensities of bands *h* and *i* are the inverse of those glucose contents: *C*. *selloana* > *A*. *donax* > *P*. *australis* (Figure 2). It is plausible that bands *h* and *i* are associated with crystalline cellulose structures, and their abundance varies inversely to that of amorphous cellulose structures. Concomitantly, given that the acid hydrolysis performed for the determination of glucose content does not hydrolyse 100% of crystalline cellulose (Torget et al., 2000), more glucose may be released from biomasses with higher amorphous cellulose content. Furthermore, band *g* (1170 – 1160 cm^–1^), which has been assigned to vibrations in glycosidic links (Table 1), is also a significantly different spectral region between the grass species. This also agrees with the interpretation that there are significant alterations in the structure of the main cell wall polysaccharides, which may be related to the crystalline arrangement of cellulose.

Lastly, bands *f* (1240 – 1235 cm^–1^) and *l* (840 – 830 cm^–1^), which have respectively been ascribed to vibrations in xylan and lignin structures, are significantly different between the stem biomass of grass species, despite not being reflected in the xylose and lignin percentage determinations made for the stem biomasses of these species (Figure 6 and Table 2). Nonetheless, these associations made for leaf and stem biomass provide strong evidence that the bands assigned according to the literature are indeed strongly correlated with the corresponding cell wall compounds in the grasses being studied.

### Mild Alkali Pretreatment and Impact on Saccharification

Alkaline pretreatments have been considered to increase the biodegradability of lignocellulosic feedstocks (Jackson, 1977; Sharma et al., 2013). Mild alkali pretreatments are known to result in a controlled de-esterification of the biomass samples, minimising lignin and carbohydrate losses (Kong et al., 1992; Chen et al., 2013; Chen et al., 2014).

To further understand the potential of the studied grass biomasses for applications in biorefining, AIR samples were treated with a mild alkali pretreatment followed by enzymatic saccharification. The alkali pretreatment had a significant effect on saccharification (*p* ≤ 0.05; Figure 5), as AIK yields were on average 2-fold higher than AIR. However, the increase varied between the species and organs (Supplementary Table 1). The most substantial effect of the alkali pretreatment was seen in *C*. *selloana*, as AIK yields were 3.3-fold higher in leaves, and 2.5-fold higher in stems. In stems of *A*. *donax* the increase was 2.7-fold in AIK samples, whereas for the remaining samples, saccharification yield increases ranged between 1- and 2-fold. In comparison to *M*. × *giganteus*, all spontaneous grass species showed higher saccharification yields.

FTIR-ATR analysis (Figure 3) showed clear differences between AIR and AIK samples at spectral regions *a* (1736 – 1730 cm^–1^; assigned to C = O stretching in acetyl-xylans) and *f* (1240 – 1235 cm^–1^; attributed to C-O vibrations of acetyl (Table 1). The intensity of these bands is reduced in pretreated biomass and is observed across all grass species. However, the de-acetylation effect appears to be more complete in stem biomass than in leaves. This is presumably due to the presence of higher amounts of secondary metabolites in leaves, given their more diversified physiological roles, when compared to stems.

It has been reported that alkaline saponification during mild alkali pretreatment is able to release acetylester substituents from heteroxylans in grass cell walls (da Costa et al., 2017). This agrees with our results, that the most noticeable and consistent compositional differences between AIR and AIK samples is seen with bands associated to xylan and acetyl substituents. These differences are the result of the loss of acetyl groups in the biomass, specifically in xylans, as *O*-acetylated xylan is the main source of acetylesters in grass cell walls (Pauly and Scheller, 2000).

Direct determination of acetylester concentrations was not performed in the present study. However, HPLC-PDA-ESI-MS^*n*^ was performed for the compositional analysis of the supernatants derived from the alkali pretreatment (Table 3). Coumaric acid, ferulic and diferulic acid isomers were observed in the liquid fractions from all alkali-pretreated biomasses. Feruloyl, diferuloyl and *p*-coumaroyl esters are abundant compounds bound to cell wall structures, namely to arabinoxylans, the main hemicellulose found in grasses (Williamson et al., 1998; Määttä-Riihinen et al., 2004). This observation corroborates that the mild alkali pretreatment promotes the hydrolysis of ester linkages, partially removing esterified substituents that may inhibit saccharification (Grohmann et al., 1986; Kong et al., 1992; Ishii, 1997; Buanafina et al., 2006; Pawar et al., 2013). Furthermore, by breaking ester bonds that cross-link polysaccharides with each other and with lignin, cellulose becomes more accessible to hydrolytic enzymes (Hendriks and Zeeman, 2009; Xu et al., 2012; Li et al., 2013; Wyman et al., 2013). The removal of ester-linked coumaric acid, which is known to be mostly bound to lignin (Sun et al., 1998; Grabber et al., 2004), may also compromise lignin structure thus deteriorating cell wall integrity. Additionally, some separation of lignin from the structural polysaccharides may be promoted, and a similar hypothesis has been presented by Paripati and Dadi (2014) as an explanation for the mechanism of action of a mild alkali treatment. Indeed, lignin content was slightly decreased in all alkali-pretreated biomass (Figure 6 and Table 2), although this was only significant in stems of *M*. *giganteus*.

It was shown that mild alkali does not cause significant loss of individual neutral monosaccharide components (Table 2). In fact, the percentages of individual monosaccharides are typically higher in alkali-pretreated samples than in non-pretreated (Figure 6), although this is likely to be due to an increase of efficiency of the acid hydrolysis. Nonetheless, it has been previously demonstrated that even low concentrations of alkali can extract pectin from grass cell walls (da Costa et al., 2017). Although it did not emerge as significantly different (*p* ≤ 0.00001), a spectral region centred at 1103 cm^–1^ showed a marked increased intensity in *C*. *selloana* stems (Figure 2). This band has been ascribed to pectic polysaccharides (Coimbra et al., 1999; Kačuráková et al., 2000; McCann et al., 2001). Pectic polysaccharides were not directly quantified in this study. However, despite grass cell walls containing low amounts of pectin (Carpita, 1996), they play important roles in maintaining structural integrity (Tan et al., 2013; Lionetti et al., 2015; Biswal et al., 2018). It is plausible that higher pectin contents could make stems from *C*. *selloana* more prone to degradation than other grass species when treated with 0.1 M NaOH. Thus partly explaining the higher saccharification yields observed in this biomass (Figure 5).

### Effects of White-Rot Fungi Pretreatments in Grass Biomasses

The relevance of using WRF-mediated biomass pretreatments is that these organisms can degrade lignin more readily than holocellulose (Valmaseda et al., 1991; Taniguchi et al., 2005; Abdel-Hamid et al., 2013; López et al., 2017). By comparing the FTIR-ATR spectra of stem biomass treated with WRF with the spectra from NF controls, it was observed that they are less affected by the fungal pretreatment than leaves (Figure 4). According to the modifications in the FTIR-ATR spectra of leaves, it is in *A*. *donax* and *P*. *australis* biomass that the most noticeable effect of the WRF treatment is observed, particularly in spectral region *b* (1625 – 1635 cm^–1^) and an adjacent band centred at 1600 cm^–1^, which have respectively been correlated with non-esterified carboxyl groups in polysaccharides (Table 1), and aromatic ring stretching (Pandey and Pitman, 2003; Bekiaris et al., 2015). This observation may be the consequence of WRF-mediated de-esterification and modification of ester-linked phenolic hydroxycinnamates involved in cell wall polymer cross-linking (Ishii, 1997; Ralph, 2010). As HPLC-PDA-ESI-MS^*n*^ was used to characterise the liquid fractions produced during the incubation with WRF, it was observed that in leaves treated with *P*. *ostreatus*, for all plant species, the liquid fractions contained ferulic and diferulic acid isomers. Whereas in stems, these compounds were detected only at trace levels, or not at all, in *A*. *donax* and in *M*. × *giganteus* (Table 3).

WRF are known to secrete a diversity of feruloyl and coumaroyl esterases (Akin et al., 1995; Linke et al., 2013; Nieter et al., 2014; Dilokpimol et al., 2016; Kelle et al., 2016) which catalyse the hydrolysis of ester bonds between ferulic and coumaric acids and plant cell wall polysaccharides. In our experiments, except for *A*. *donax* stems, in all biomass treated with WRF, ferulic or diferulic acid isomers were released. By contrast, coumaric acid was only detected at trace levels in *P*. *australis* stems, not being found in any other biomass. This observation agrees with reports from other authors who have demonstrated that ferulic acid primarily, but also coumaric acid, are removed in grass biomass treated with WRF, breaking cell wall cross-links and improving saccharification (Agosin et al., 1985; Valmaseda et al., 1991).

In the saccharification assay performed in this study, although the differences in relation to the controls were statistically significant in few cases, further interesting inferences can be drawn by interpreting the absolute values. The saccharification yield of *A*. *donax* leaf biomass was increased when incubated with *P*. *ostreatus*, while in stems, these yields were increased in *A*. *donax*, *C. selloana* and *P. australis*, when treated with *G. lucidum*. In *M*. × *giganteus* stem, when treated with *T. versicolor*, saccharification yield increased in relation to the control. However, in the remaining cases, when samples are treated with WRF, the saccharification yields were lower than the controls (Figure 5 and Supplementary Table 1). This is presumably due to the release of enzyme-inhibitory compounds during fungal action. It is known that pretreatment-derived soluble compounds can inhibit saccharification, via steric hindrance for binding of hydrolytic enzymes (Biely, 2012; Pawar et al., 2013; Zhai et al., 2018). To address this issue and enhance pretreatment efficiency, a mild alkali pretreatment was employed (0.1 M NaOH; 24 h; 21°C), which substantially increased saccharification yields in relation to their non-alkali treated counterparts. Once again, looking at the absolute values, in comparison to the controls, in some cases, the results suggest that a synergistic effect does indeed occur when the WRF and mild alkali pretreatments are combined. Namely, in *C*. *selloana* leaves treated with alkali and *P. ostreatus*, the saccharification yield was 637.9 nmol mg^–1^, which is higher than the samples treated only with alkali (615.1 nmol mg^–1^), and almost 2-fold higher than the non-pretreated controls (359.0 nmol mg^–1^). In stems treated with alkali subsequently to the WRF pretreatment (Figure 5 and Supplementary Table 1), similar relationship can be established for: *A. donax* treated with *G. lucidum*, *T. versicolor* and *P. ostreatus*; *C. selloana* treated with *T. versicolor* and *P. ostreatus*; and *M*. × *giganteus* treated with *T. versicolor*. In all these cases the saccharification yield of biomass treated with a combination of WRF and the mild alkali pretreatment was higher than that of samples treated only with alkali and, than non-pretreated controls. Furthermore, in all these combinations, lignin content in pretreated samples is lower than in non-pretreated controls (Table 2 and Figure 5). It is likely that some lignin loss, detachment, or structural alteration is responsible for the increase seen in saccharification.

Under the tested conditions it cannot be excluded that a synergistic effect of combined WRF-ALK pretreatments is responsible for a disruption of cell wall integrity. These modifications are likely to be at the level of cell wall polymers, namely in lignin, and in ester-bound substituents, such as ferulic acid. Ultimately, a structurally compromised cell wall would be more susceptible to hydrolysis by cellulolytic enzymes, leading to increased saccharification yields.

### Conclusions and Final Remarks

*Arundo donax*, *C*. *selloana* and *P*. *australis* are grass species that grow spontaneously throughout Southern Europe, including Portugal. By characterising their biomass, potential applications can be proposed, contributing to their economic valorisation.

Some compositional variation will be expected between individuals due to heterozygosity of wild populations. Nonetheless, as the aim of this study was to probe the potential value of wild-grown biomass from various species, a compromise had to be made in terms of the number of replicates. When compared to *M*. × *giganteus* composition, a potential lignocellulosic crop, the three spontaneous grass species showed higher xylose content, higher glucose in *P*. *australis* and lower lignin in *C*. *selloana* and *P*. *australis*. Furthermore, saccharification yields are higher in *A*. *donax*, *C*. *selloana* and *P*. *australis*, than in *M*. × *giganteus*. Our results suggest that the biomass from spontaneous grasses has a comparable biorefining potential as *M*. × *giganteus*. To this, there is an added advantage that *A*. *donax*, *C*. *selloana* and *P*. *australis* occur spontaneously on marginal lands; which mostly consist of lands that have been abandoned due to relocation of agriculture, low productivity, or with physical or environmental constraints to agriculture (Daily, 1995; Campbell et al., 2008; Cai et al., 2011; Dauber et al., 2012; Pancaldi and Trindade, 2020). Considering these observations, future studies will be drawn to assess how the heterozygosity of the biomass affects valorisation potential across a wild population and between different geographic origins.

Lignocellulosic biomass from high-biomass-producing grasses, such as those studied here, can be a raw material to produce a wide range of industry-relevant products. However, biorefinery of plant biomass is limited by cell wall recalcitrance. To address this issue, this study also involved the application of fungal pretreatments, with or without a combination with mild alkali, followed by saccharification, and pretreatment liquid fraction analysis. This study has contributed to the characterisation of the mechanism of action of the employed pretreatments. In the biomass of the grass species being studied, both the WRF and the mild alkali approaches seem to act mainly by de-esterification of the biomass, breaking crosslinks between the cell wall polymers, thus increasing its porosity and allowing better access to hydrolytic enzymes. Significant amounts of coumaric, ferulic and diferulic acid, among other compounds, were released during the WRF and mild alkali pretreatments. Furthermore, a possible synergistic effect was also revealed, as the effect of combining the alkali and the WRF pretreatments produced higher saccharification yields than a given pretreatment on its own.

One of the aims of lignin-first approaches is to obtain fewer but uniform products from biomass fractionation through the application of milder pretreatments (Korányi et al., 2020). This study suggests that fractionation approaches where combinations of WRF and mild alkali are used may represent a strategy for controlled depolymerisation of lignin. However, further work is required to optimise these methodologies.

This work represents the first study where the biorefining potential of spontaneous *A*. *donax*, *P*. *australis* and *C*. *selloana* from marginal lands is assessed in comparison to a trial field-grown lignocellulose-dedicated crop such as *M*. × *giganteus*. These spontaneous grasses were previously uncharacterised in a biorefining context. Thus, compositional characterisation data generated here will contribute to the advancement of novel lignocellulosic crops and opportunities to valorise these resources. This, in turn, may contribute to industry and create capital, as a new economic crisis is arising. Additionally, the potential added value to these spontaneous grass species may create a monetary incentive for voluntary biomass culling by landowners, thus contributing to reduce excessive biomass accumulation in marginal and unused lands, providing new uses for these areas and vegetation.

## Data Availability Statement

The raw data supporting the conclusions of this article will be made available by the authors, without undue reservation.

## Author Contributions

RC planned and designed the research and performed experiments and data analyses. High-throughput saccharification assays were performed at the Centre for Novel Agricultural Products (CNAP) at the University of York (UK), in collaboration with RS and LG. HPLC-PDA-ESI-MS^*n*^ and HPAEC-PAD analyses were performed at the Institute of Biology, Environmental and Rural Sciences (IBERS) at Aberystwyth University (UK), in collaboration with AW, BH, and MB. The manuscript was produced by RC, with critical feedback from all co-authors. All authors contributed to the article and approved the submitted version.

## Conflict of Interest

The authors declare that the research was conducted in the absence of any commercial or financial relationships that could be construed as a potential conflict of interest.

## Supplementary Material

Supplementary Figure 1 Correlation matrix between the spectral bands of interest most significantly different FTIR-ATR spectral regions between the different grass species (cm^–1^): (*a*, 1736 – 1730; *b*, 1625 – 1635; *c*, 1515 – 1505; *d*, 1322 – 1310; *e*, 1275 – 1256; *f*, 1240 – 1235; *g*, 1170 – 1160; *h*, 1060 – 1055; *i*, 1035 – 1030; *j*, 993 – 985; *k*, 898 – 890; *l*, 840 – 830). See main text and Table 1 for more information.

Supplementary Table 1 Saccharification of leaf and stem biomass (nmol mg^–1^) from four poalean species measured by a high-throughput saccharification assay after 8 h incubation in a hydrolytic enzyme mixture. See main text for more information. Acronyms: AIR, alcohol insoluble residue; AIK, alcohol insoluble residue treated with a 0.1 M NaOH mild alkali pretreatment; NF, no fungi; GAN, *Ganoderma lucidum*; PLE, *Pleurotus ostreatus*; TRA, *Trametes versicolor*. Values are expressed as mean ± standard error.

Supplementary Table 2 Percentage of recovered biomass after white-rot fungi (WRF; 30-day incubation) and mild alkaline 0.1 M NaOH for 24 h at 21°C (ALK) pretreatments.
