# Supplementary figures and images for: Biorefining Potential of Wild-Grown Arundo donax, Cortaderia selloana and Phragmites australis and the Feasibility of White-Rot Fungi-Mediated Pretreatments
Source: Front Plant Sci. 2021 Jul 2;12:679966. doi: 10.3389/fpls.2021.679966 (PMC8283202; doi:10.3389/fpls.2021.679966)

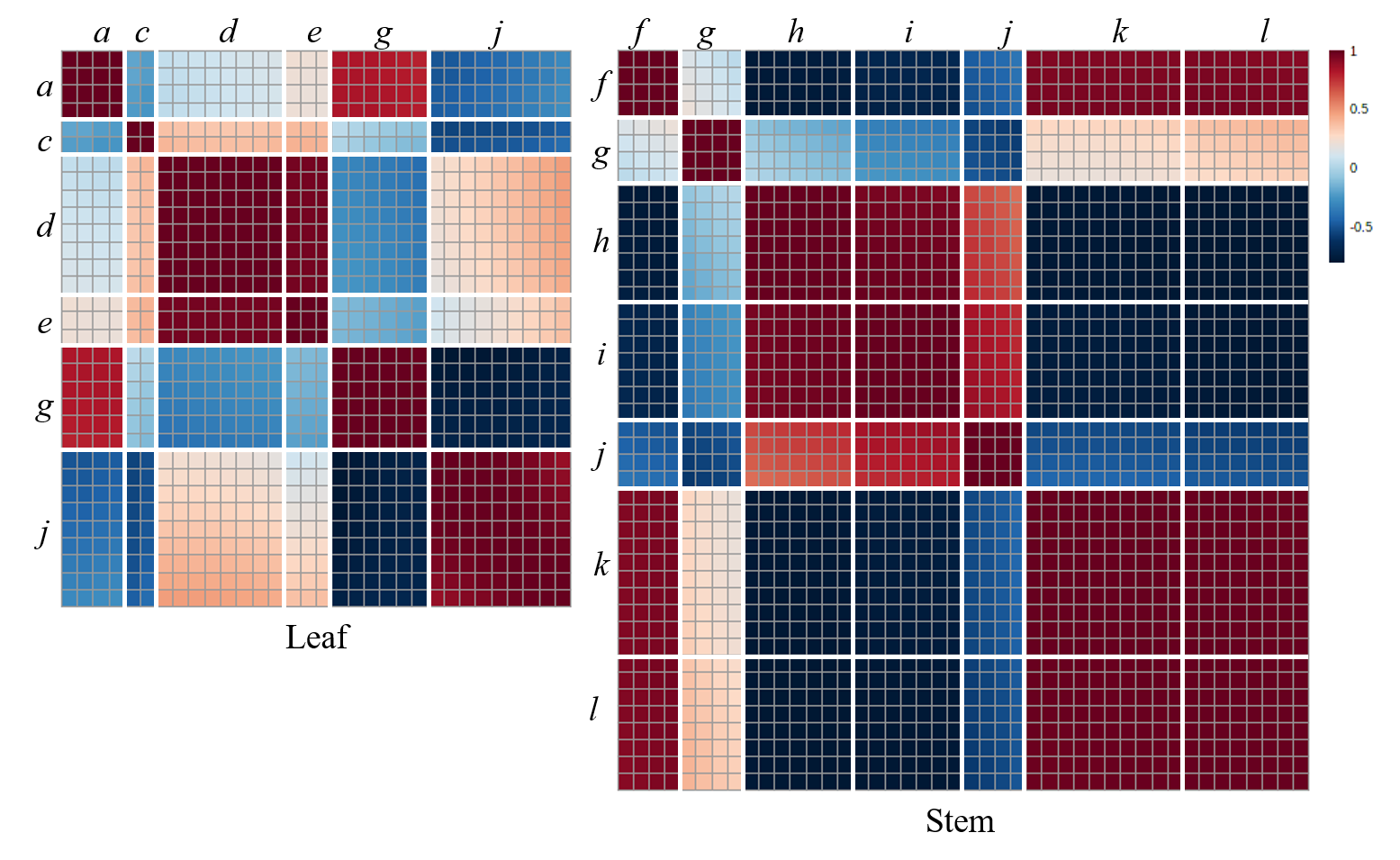

Supplement: Supplementary Figure 1 — Correlation matrix between the spectral bands of interest most significantly different FTIR-ATR spectral regions between the different grass species (cm–1): (a, 1736 – 1730; b, 1625 – 1635; c, 1515 – 1505; d, 1322 – 1310; e, 1275 – 1256; f, 1240 – 1235; g, 1170 – 1160; h, 1060 – 1055; i, 1035 – 1030; j, 993 – 985; k, 898 – 890; l, 840 – 830). See main text and Table 1 for more information. [file Image_1.TIF]
