# Supplementary material for: Biorefining Potential of Wild-Grown Arundo donax, Cortaderia selloana and Phragmites australis and the Feasibility of White-Rot Fungi-Mediated Pretreatments
Source: Front Plant Sci. 2021 Jul 2;12:679966. doi: 10.3389/fpls.2021.679966 (PMC8283202; doi:10.3389/fpls.2021.679966)
Supplement: Supplementary Table 1 — Saccharification of leaf and stem biomass (nmol mg–1) from four poalean species measured by a high-throughput saccharification assay after 8 h incubation in a hydrolytic enzyme mixture. See main text for more information. Acronyms: AIR, alcohol insoluble residue; AIK, alcohol insoluble residue treated with a 0.1 M NaOH mild alkali pretreatment; NF, no fungi; GAN, Ganoderma lucidum; PLE, Pleurotus ostreatus; TRA, Trametes versicolor. Values are expressed as mean ± standard error. [file Table_1.DOCX]

| **Table S1.** Saccharification of leaf and stem biomass (nmol·mg^-1^) from four poalean species measured by a high-throughput saccharification assay after 8h incubation in a hydrolytic enzyme mixture. See main text for more information. Acronyms: AIR, alcohol insoluble residue; AIK, alcohol insoluble residue treated with a 0.1M NaOH mild alkali pretreatment; NF, no fungi; GAN, G*anoderma lucidum*; PLE, *Pleurotus ostreatus*; TRA, *Trametes versicolor*. Values are expressed as mean ± standard error. | | | | | | | | | | | | | | | | | |
| --- | --- | --- | --- | --- | --- | --- | --- | --- | --- | --- | --- | --- | --- | --- | --- | --- | --- |
|  |  | **Leaf** | | | | | | |  | **Stem** | | | | | | |  |
|  |  | **AIR** |  | **AIK** |  |  |  |  |  | **AIR** |  | **AIK** |  |  |  |  |  |
| ***Arundo donax*** |  | 440.4 ± 21.3 |  | 799.2 ± 41.2 |  |  |  |  |  | 244.5 ± 5.2 |  | 671.5 ± 20.7 |  |  |  |  |  |
| ***Cortaderia selloana*** |  | 202.0 ± 5.6 |  | 675.1 ± 25.8 |  |  |  |  |  | 328.0 ± 7.4 |  | 812.1 ± 16.2 |  |  |  |  |  |
| ***Phragmites australis*** |  | 541.8 ± 38.6 |  | 689.9 ± 41.0 |  |  |  |  |  | 375.2 ± 11.8 |  | 645.2 ± 10.5 |  |  |  |  |  |
| ***Miscanthus × giganteus*** |  | 298.3 ± 13.3 |  | 525.5 ± 8.4 |  |  |  |  |  | 248.8 ± 14.8 |  | 322.4 ± 8.4 |  |  |  |  |  |
|  |  |  |  |  |  |  |  |  |  |  |  |  |  |  |  |  |  |
|  |  | **NF** |  | **GAN** |  | **PLE** |  | **TRA** |  | **NF** |  | **GAN** |  | **PLE** |  | **TRA** | **No alkali** |
| ***Arundo donax*** |  | 398.4 ± 16.9 |  | 343.6 ± 24.1 |  | 503.7 ± 21.5 |  | 198.9 ± 2.7 |  | 394.6 ± 16.5 |  | 410.6 ± 9.9 |  | 240.6 ± 3.6 |  | 326.9 ± 14.1 |  |
| ***Cortaderia selloana*** |  | 359.0 ± 11.6 |  | 317.5 ± 12.3 |  | 187.5 ± 5.6 |  | 264.8 ± 7.4 |  | 485.7 ± 33.5 |  | 485.5 ± 6.3 |  | 387.8 ± 15.8 |  | 435.5 ± 28.6 |  |
| ***Phragmites australis*** |  | 470.0 ± 25.3 |  | 280.9 ± 10.1 |  | 421.4 ± 27.4 |  | 412.1 ± 5.6 |  | 473.5 ± 18.9 |  | 479.7 ± 17.8 |  | 184.1 ± 9.3 |  | 360.3 ± 9.5 |  |
| ***Miscanthus × giganteus*** |  | 311.7 ± 21.5 |  | 173.4 ± 4.9 |  | 147.4 ± 3.4 |  | 156.8 ± 5.8 |  | 213.2 ± 3.3 |  | 101.6 ± 0.5 |  | 108.8 ± 4.8 |  | 331.1 ± 3.1 |  |
|  |  |  |  |  |  |  |  |  |  |  |  |  |  |  |  |  |  |
|  |  | **NF** |  | **GAN** |  | **PLE** |  | **TRA** |  | **NF** |  | **GAN** |  | **PLE** |  | **TRA** | **Alkali** |
| ***Arundo donax*** |  | 612.4 ± 14.4 |  | 579.9 ± 11.3 |  | 465.5 ± 10.4 |  | 317.5 ± 5.3 |  | 461.3 ± 18.0 |  | 589.4 ± 26.3 |  | 486.9 ± 11.1 |  | 528.1 ± 23.1 |  |
| ***Cortaderia selloana*** |  | 615.1 ± 23.8 |  | 536.4 ± 17.9 |  | 637.9 ± 12.9 |  | 449.5 ± 21.6 |  | 658.9 ± 19.4 |  | 602.6 ± 26.3 |  | 725.2 ± 21.9 |  | 674.4 ± 19.0 |  |
| ***Phragmites australis*** |  | 518.2 ± 17.4 |  | 426.4 ± 20.5 |  | 407.8 ± 9.6 |  | 525.1 ± 8.8 |  | 578.6 ± 17.6 |  | 534.6 ± 26.0 |  | 458.9 ± 5.0 |  | 547.6 ± 18.0 |  |
| ***Miscanthus × giganteus*** |  | 667.5 ± 3.7 |  | 398.8 ± 1.8 |  | 372.7 ± 11.4 |  | 188.9 ± 8.6 |  | 319.3 ± 10.0 |  | 246.8 ± 11.1 |  | 163.2 ± 4.3 |  | 539.4 ± 32.5 |  |
