# Supplementary material for: Biorefining Potential of Wild-Grown Arundo donax, Cortaderia selloana and Phragmites australis and the Feasibility of White-Rot Fungi-Mediated Pretreatments
Source: Front Plant Sci. 2021 Jul 2;12:679966. doi: 10.3389/fpls.2021.679966 (PMC8283202; doi:10.3389/fpls.2021.679966)
Supplement: Supplementary Table 2 — Percentage of recovered biomass after white-rot fungi (WRF; 30-day incubation) and mild alkaline 0.1 M NaOH for 24 h at 21°C (ALK) pretreatments. [file Table_2.DOCX]

**Table S2.** Percentage of recovered biomass after white-rot fungi (WRF; 30-day incubation) and mild alkaline 0.1M NaOH for 24h at 21 °C (ALK) pretreatments.

|  |  |  |  | **WRF*-pretreated** |  | **ALK-pretreated** |  | **WRF* + ALK pretreated** |
| --- | --- | --- | --- | --- | --- | --- | --- | --- |
|  |  |  |  |  |  |  |  |  |
| **Leaf** |  | *Arundo donax* |  | 97.0% |  | 96.2% |  | 93.7% |
|  |  | *Cortaderia selloana* |  | 97.8% |  | 97.1% |  | 95.3% |
|  |  | *Phragmites australis* |  | 96.5% |  | 95.5% |  | 92.6% |
|  |  | *Miscanthus × giganteus* |  | 98.0% |  | 97.7% |  | 96.9% |
|  |  |  |  |  |  |  |  |  |
| **Stem** |  | *Arundo donax* |  | 97.7% |  | 97.0% |  | 95.1% |
|  |  | *Cortaderia selloana* |  | 94.9% |  | 93.4% |  | 89.2% |
|  |  | *Phragmites australis* |  | 97.8% |  | 97.1% |  | 95.3% |
|  |  | *Miscanthus × giganteus* |  | 98.0% |  | 97.5% |  | 95.9% |
|  |  |  |  |  |  |  |  |  |
| * Based on *Pleurotus ostreatus* pretreatment. | | | | |  |  |  |  |
